# Association Between Gum Chewing and Temporomandibular Disorders

**DOI:** 10.3390/jcm14155253

**Published:** 2025-07-24

**Authors:** Yana Yushchenko, Michał Zemowski, Daniil Yefimchuk, Aneta Wieczorek

**Affiliations:** 1 Student Research Group, Department of Prosthodontics and Orthodontics, Faculty of Medicine, Jagiellonian University Medical College, 31-155 Krakow, Poland; yana.yushchenko39@gmail.com (Y.Y.); m.zemowski@gmail.com (M.Z.); 2 Private Practice, 30-699 Krakow, Poland; thechanging99@gmail.com; 3 Department of Prosthodontics and Orthodontics, Institute of Dentistry, Faculty of Medicine, Jagiellonian University Medical College, 31-155 Krakow, Poland

**Keywords:** temporomandibular disorders, temporomandibular joint, DC/TMD, gum chewing, parafunctional habits, oral behaviors, bruxism, stress, young adults, medical students

## Abstract

**Background**: Gum chewing is a common habit among young adults, often promoted for its oral health and psychological benefits. However, as a repetitive and non-functional activity, it is also considered a potential risk factor for temporomandibular disorder (TMD), particularly when practiced chronically. The aim of this study was to evaluate whether excessive gum chewing is associated with a higher prevalence of TMD among young adults presumed to be under elevated academic stress based on their demographic characteristics. **Methods**: Participants were examined in Krakow, Poland, using the Diagnostic Criteria for Temporomandibular Disorders (DC/TMD) protocol. Participants completed a structured questionnaire assessing gum-chewing frequency, duration, and chronicity. Associations between chewing behaviors and TMD presence were analyzed using univariate logistic regression (α = 0.05). **Results**: This study included young adults 66 participants aged 19–30. TMD was diagnosed in 55 participants (83.3%), including muscular disorders (n = 9; 16.4%), articular disorders (n = 10; 18.2%), and combined muscular–articular disorders (n = 38; 57.6%). More than 70% of participants reported chewing gum for over five years. No statistically significant associations were found between TMD occurrence and the frequency, duration, or chronicity of gum chewing (*p* > 0.05). **Conclusions**: These findings suggest that, in the absence of other contributing factors, gum chewing may not independently contribute to TMD development. The elevated TMD prevalence may reflect confounding variables such as high academic stress, narrow age distribution, or female predominance. However, the limited sample size limits statistical power, particularly for detecting subtle effects potentially distorted by other variables. Additionally, the cross-sectional nature of this study precludes causal interpretation. Further studies in larger and more heterogeneous populations are recommended.

## 1. Introduction

### 1.1. Masticatory Function

Chewing is a fundamental function of the stomatognathic system, responsible for mechanical food breakdown and salivary stimulation. However, neuroimaging studies suggest that its effects extend beyond digestion—mastication activates a widespread network of brain regions and enhances cerebral blood flow [1]. These activations intensify when chewing involves greater intensity and richer flavor, or occurs under emotional stress [1,2]. This evidence illustrates how both the mechanical and sensory aspects of mastication influence neural responses. Mastication also promotes the release of neurotransmitters involved in regulating attention, mood, and memory [3,4]. Regular chewing has been associated with preserved cognitive function through mechanisms that support neuroplasticity and reduce stress [5,6,7]. However, the long-term behavioral consequences of habitual chewing—particularly in the context of stress modulation or cognitive adaptation—remain unclear and warrant further longitudinal investigation.

### 1.2. Gum Chewing as a Parafunction

Chewing gum is a common habit among adolescents and young adults. Its widespread use has been attributed to its diverse applications—it may support oral hygiene by stimulating salivary production [8]. However, despite its potential benefits [9,10], chewing gum is classified as a parafunctional habit in the Diagnostic Criteria for Temporomandibular Disorders (DC/TMD) [11,12], which raises concerns about its long-term effects on the stomatognathic system.

Parafunctional habits refer to repetitive non-functional oral behaviors that exceed normal physiological demands and may lead to musculoskeletal strain or joint overload. In the case of gum chewing, such effects are particularly relevant when mastication occurs excessively or without nutritional purpose. Repetitive chewing—especially when performed unconsciously or asymmetrically—may expose the masticatory system to sustained low-load mechanical stress, potentially contributing to myofascial pain, limited mandibular mobility, joint sounds, or tension-type headaches in individuals with predisposing factors such as stress or malocclusion [13].

Although it has been demonstrated that healthy individuals typically recover fully after prolonged chewing, chronic overuse may still induce microtrauma or exacerbate pre-existing dysfunctions [13,14]. These patterns can disrupt muscle coordination, overload the temporomandibular joints (TMJs), and lead to functional disturbances such as articular displacement or pain [13,14,15].

Recent evidence underscores the complexity of the relationship between gum chewing and temporomandibular disorder (TMD). Several studies have reported an association between gum chewing and increased jaw muscle discomfort [16,17]. In contrast, other studies described only transient symptoms that resolved after discontinuing gum use [13,18]. These mixed findings highlight the need to better understand how habitual behaviors such as gum chewing relate to the broader context of TMDs.

### 1.3. TMD: Prevalence, Causes, and Diagnosis

Epidemiological studies indicate that TMDs are among the most prevalent musculoskeletal conditions affecting the craniofacial region. The newest research estimates the global prevalence of TMD at 34%, highlighting the substantial burden of this condition in the general population. However, the occurrence of TMD varies considerably across continents and age groups [19]. Women consistently demonstrate a higher prevalence and greater severity of TMD [20,21]. Research indicates that gender differences in orofacial pain not only persist but may widen over time, with females experiencing both increased incidence and less favorable recovery trajectories [21].

TMDs are widely recognized as multifactorial conditions influenced by anatomical, behavioral, and psychosocial factors [22]. Behavioral contributors—particularly oral parafunctions such as bruxism, clenching, and gum chewing—are consistently associated with the onset and persistence of TMD symptoms [14,23]. These behaviors are significant predictors, especially when performed frequently or asymmetrically [14]. Their habitual nature may lead to microtrauma, muscular fatigue, and, over time, joint dysfunction in predisposed individuals [13,14]. Anatomical factors such as malocclusion have traditionally been considered risk factors; however, more recent studies suggest no strong correlation between malocclusion and TMD severity [24,25], underscoring the need for a broader multifactorial perspective. Psychosocial factors also play a critical role in TMD pathogenesis. High levels of stress, anxiety, and depression are associated with increased muscle tension, altered pain perception, and reduced coping capacity. These factors are particularly relevant in patients with myofascial pain [24]. Moreover, some findings suggest that the impact of oral habits on TMD may be mediated or amplified by psychosocial distress [26]. Additional factors under investigation include vitamin D deficiency, which may impair bone metabolism, muscle function, and inflammatory regulation. One study proposed a mechanistic link between low vitamin D levels and increased TMD risk [27]. Altogether, the current evidence reinforces the biopsychosocial model of TMD, in which biological predispositions, behavioral habits, and psychological stressors interact dynamically. Several authors emphasize that the effective management of TMD should address this complex interplay rather than focus solely on occlusal or isolated anatomical factors [28,29,30].

Diagnosing TMDs remains a clinical challenge due to their nonspecific, overlapping, and often diffuse symptoms [31]. Patients commonly report pain in the jaw, temples, or ears; joint noises; restricted jaw mobility; and headaches—manifestations that may resemble various orofacial or musculoskeletal disorders [32]. This symptom variability complicates differential diagnosis, particularly in non-specialist settings. Considering the high prevalence of TMD [19] and its negative impact on quality of life—such as sleep disturbances, reduced life satisfaction, and psychosocial burden [32,33]—timely and accurate diagnosis is essential [34]. Failure to recognize and manage TMD can lead to delayed treatment and increased risk of chronic symptoms. To improve diagnostic reliability, the DC/TMD protocol was developed as a standardized tool for use in both clinical and research settings [11,12]. The DC/TMD system categorizes TMD into three primary groups: muscle disorders, joint disorders, and headache attributed to TMD. Diagnosis is based on clinical examination and validated psychosocial assessment tools. Now considered the gold standard in TMD diagnostics, the DC/TMD framework improves diagnostic consistency, supports individualized treatment, and enhances our understanding of the multifactorial nature of TMD.

### 1.4. Aim of This Study

Most previous research has focused on short-term or experimental conditions, with limited data available on the long-term real-life impact of oral behaviors on TMD symptoms. Despite widespread gum use among young adults, studies employing standardized DC/TMD diagnostic protocols to assess its association with TMD are limited, and findings remain inconclusive. This study aims to address that gap by evaluating whether gum chewing is associated with the prevalence of TMD in a sample of young adults. Medical students were chosen as the study population primarily for convenience, but also due to their known exposure to elevated academic stress, making them a relevant group for TMD-related investigations [35,36]. Given the growing popularity of chewing gum as a lifestyle habit and stress coping mechanism, especially in academic settings, it is important to assess whether its chronic use poses a risk to the masticatory system [37]. Clarifying this relationship may support more accurate patient education and prevention strategies in clinical practice, particularly among younger individuals, where parafunctional behaviors often go unnoticed or underestimated [38]. Based on the previous literature, we hypothesized that excessive gum chewing is associated with an increased prevalence of TMD among young adults.

## 2. Materials and Methods

### 2.1. Ethics Approval

Ethics approval was obtained from the Jagiellonian University Bioethics Committee (decision no. 1072.6120.16.2024) on 17 April 2024. This study was conducted in accordance with the Declaration of Helsinki and Good Clinical Practice (GCP) guidelines.

### 2.2. Study Population

This study included young adults (18–30 years) recruited from medical, dental, and physiotherapy faculties in Krakow. The age range was selected to reflect the typical age distribution of university students, which constituted the target population. Recruitment was conducted using a convenience sampling approach by posting announcements in student social media groups. Due to the observational cross-sectional design and logistical constraints, all eligible individuals available during the recruitment period were included, representing the maximum feasible sample size. All participants provided written informed consent prior to enrollment.

Inclusion criteria: Participants were eligible if they were aged 18–30 years, had complete dental arches, and provided informed consent

Exclusion criteria: Participants were excluded if they had prosthetic restorations, a previous diagnosis of TMD, significant malocclusion (Angle Class II or Class III), musculoskeletal disorders (e.g., rheumatoid arthritis, osteoarthritis, fibromyalgia), or pathological tooth mobility (Grade III according to Kantorowicz) [11,12,39].

### 2.3. Study Design

This study consisted of two stages. In the first stage, participants completed an original questionnaire assessing their gum-chewing habits, including frequency, duration, and chronicity. The questionnaire was developed specifically for this study and was pretested on members of our student research group prior to data collection. In the second stage, a clinical examination was conducted according to the DC/TMD protocol [11,12], including both questionnaire-based and physical assessment components. The clinical examinations were performed by two trained and jointly calibrated examiners at the Department of Prosthodontics, Jagiellonian University Medical College, Krakow, Poland. The examiners underwent internal training and calibration prior to data collection, sharing the same academic background and institutional environment. Although several patients were examined collaboratively to ensure consistency in diagnostic procedures, no formal assessment of inter-rater reliability was conducted. Diagnoses were established collaboratively based on the results of clinical examinations and questionnaires, ensuring a comprehensive assessment of each participant, according to DC/TMD protocol.

### 2.4. Questionnaire Content

Participants completed a structured questionnaire designed specifically for this study. The survey included items related to gum-chewing habits, with a focus on three key behavioral parameters: frequency, duration, and chronicity. Respondents were asked how often they chew gum (e.g., several times a day, once a day, several times a week, once a week, several times a month, once a month, or less frequently), how long a typical chewing session lasts (<10 min, 10–30 min, or >30 min), and how many years they have maintained the habit (<1 year, 1–3 years, 3–5 years, or >5 years). These questions allowed for the categorization of participants into subgroups for further statistical analysis, as described below.

### 2.5. Variable Classification

Participants were categorized into analytical subgroups based on their self-reported gum-chewing behaviors collected via the original questionnaire.

Chewing frequency was classified into two main categories. Individuals who reported chewing gum several times a week, once a day, or several times a day were considered systematic users, whereas those who reported chewing once a week, several times a month, once a month, or less frequently were classified as non-systematic users.

Chewing duration was defined as the typical length of a single chewing session. Participants who reported chewing for less than 10 min were categorized as short-duration chewers, while those who chewed for 10 to 30 min or more than 30 min per session were grouped as long-duration chewers. This threshold was based on published data indicating that a typical meal for adolescents lasts approximately 10 min [40]

To assess the potential cumulative exposure to masticatory load, participants were also categorized according to habit chronicity. Those who had maintained the chewing habit for up to five years were considered to have a short-term habit, whereas those reporting more than five years of chewing history were categorized as long-term users.

### 2.6. Statistical Analysis

All statistical analyses were performed using the R statistical language (version 4.4.3; R Core Team, Vienna, Austria, 2023) on Windows 10 Pro 64-bit (build 19045). No additional R packages were used. Descriptive statistics were computed for all variables. For continuous data, results are presented as mean, standard deviation (SD), median, interquartile range (IQR), minimum, and maximum. For categorical variables, absolute and relative frequencies were reported.

To examine the association between gum-chewing behaviors and the presence of TMD, univariate logistic regression models were applied. The presence of any TMD—regardless of subtype—was treated as a binary outcome (TMD vs. no TMD). Overlapping diagnoses were included under the general “TMD” category. Each behavioral parameter—chewing frequency, duration, and chronicity—was analyzed independently. Results are expressed as odds ratios (ORs) with corresponding 95% confidence intervals (CIs) and *p*-values. A significance threshold of α = 0.05 was adopted. Effect sizes were interpreted according to the thresholds: small (OR = 1.68), medium (OR = 3.47), and large (OR = 6.71), proposed by Chinn (2000) [41].

## 3. Results

A total of 66 participants met the inclusion criteria. They were aged between 19 and 30 years (mean 23.44 ± 1.72; median 23, IQR: 23–24), and the majority were female (n = 41; 62.1%). Characteristics of the study group are presented in Table 1.

TMD was diagnosed in 55 participants (83.3%) using the DC/TMD protocol. Muscle-related disorders were found in 9 participants (13.6%), joint-related in 14 (22.7%), and combined muscle-joint disorders in 32 (47.0%). Eleven individuals (16.7%) were classified as TMD-free.

A descriptive comparison by gender revealed a slightly higher prevalence of TMD among female participants (35 out of 41; 85.4%) compared with males (20 out of 25; 80.0%). Combined disorders were the most common diagnosis in both groups (48.8% in females, 48.0% in males). Muscle-related disorders such as myalgia were more frequent among females (n = 7) than males (n = 2), while joint-related TMD occurred with comparable frequency (8 vs. 6 cases, respectively). The distribution of TMD subtypes by gender is summarized in Table 2.

A more detailed breakdown of bilateral TMD diagnoses is presented in Table 3 and Table 4, covering muscle and joint disorders, respectively.

Clinical examinations were performed bilaterally for each participant, resulting in a total of 132 assessed sides. Among muscle-related disorders, myalgia was the most frequently diagnosed condition (n = 18), followed by a single case of arthralgia. In the domain of joint-related disorders, disc displacement with reduction was the most common finding (n = 19), while disc displacement without reduction was diagnosed in seven participants—one with limited opening and six without. Degenerative joint disease was observed in one case. The category “other diagnosis” refers to cases where clinical signs and symptoms of TMD were observed but the findings did not fulfill the formal diagnostic criteria for specific DC/TMD categories (e.g., due to insufficient criteria or atypical presentation). It is important to note that multiple diagnoses were often present simultaneously, and the total number of reported diagnoses exceeded the number of participants due to bilateral assessment and overlapping conditions.

Based on responses to the original questionnaire on gum-chewing habits, participants were divided into subgroups by chewing frequency, duration per session, and chronicity of the habit. Systematic gum use was reported by 29 individuals (43.9%), while 37 (56.1%) were classified as non-systematic chewers. Regarding chewing duration, 29 participants (43.9%) reported sessions lasting less than 10 min, 33 (50%) chewed for 10–30 min, and 4 (6.1%) reported sessions exceeding 30 min. Long-term gum chewing, defined as more than 5 years of habit persistence, was reported by 47 participants (71.2%). The detailed distribution of participants is presented in Table 5.

Univariate logistic regression analysis revealed no statistically significant associations between chewing frequency, chewing duration, or chronicity of the habit and the presence of TMD (*p* > 0.05 for all parameters). Odds ratios and 95% confidence intervals for each variable are presented in Table 6. All odds ratios were below 1.68, indicating small effect sizes according to Chinn [41].

## 4. Discussion

### 4.1. Main Findings

The aim of this study was to investigate whether excessive gum chewing is associated with an increased prevalence of TMD among young adults. Although 83.3% of participants were diagnosed with TMD, univariate logistic regression analysis revealed no statistically significant associations between TMD diagnosis and any of the chewing-related variables—frequency, duration, or chronicity (all *p* > 0.05).

Moreover, based on the effect size thresholds proposed by Chinn (2000) [41], the observed odds ratios (ORs < 1.68) indicate only small effects, suggesting limited predictive value of these behaviors when considered in isolation. However, it is important to emphasize that the lack of statistical significance does not necessarily rule out a meaningful association. For example, the OR of 1.55 for prolonged gum chewing may indicate a subtle trend that did not reach significance, potentially due to the limited statistical power of the current sample.

### 4.2. High Prevalence of TMD

The prevalence of TMD in our study group (83.3%) was considerably higher than the global average of approximately 34% in the general population reported in a most recent meta-analysis [19]. However, the wide range of TMD prevalence reported in the literature—from 5% to 88%, depending on diagnostic criteria and population characteristics [42]—suggests that such a discrepancy is not unusual and requires careful interpretation.

Several factors likely contributed to the elevated rate observed in our study. First, the sample consisted exclusively of medical students aged 19–30, a demographic known to experience high levels of academic stress. Psychosocial stress has been repeatedly associated with muscular TMD symptoms, particularly myalgia. Academic pressure, irregular sleep, and performance-related anxiety may amplify parafunctional behaviors, such as clenching or gum chewing, which further increase susceptibility to TMD [43,44].

Second, sex-based differences may have influenced the results. Although no statistical analysis of sex-related differences was performed, descriptive data revealed a slightly higher TMD prevalence in females (85.4%) compared with males (80.0%). Previous studies have shown that women are more susceptible to TMD, often reporting greater pain intensity and poorer recovery outcomes [20,21]. This disparity may also be influenced by hormonal modulation of pain, as well as psychosocial and behavioral factors. The female predominance in our sample (62.1%) may therefore have slightly inflated the overall prevalence [45].

Third, individuals who suspected they had TMD and were interested in receiving a diagnosis may have been more likely to participate, further inflating the observed prevalence. Moreover, the DC/TMD protocol is characterized by high sensitivity and specificity, ensuring the reliable detection of TMD subtypes. However, its thoroughness may also partly explain the elevated number of positive diagnoses observed in our study [11,12]. All of these factors should be taken into account when assessing the broader applicability of our findings.

### 4.3. Gum Chewing as a Modulating Factor

TMDs are acknowledged as conditions with multifaceted origins, involving a dynamic interaction between anatomical structures, behavioral patterns, and psychosocial influences [22]. In this multifactorial context, gum chewing may serve not as a primary cause but as a contributing factor—unlikely to provoke dysfunction in isolation, yet potentially relevant when combined with other risk modifiers such as elevated stress, bruxism, or occlusal discrepancies [28]. This interpretation is consistent with our findings, which showed no consistent dose–response association between gum use and TMD prevalence, despite widespread and prolonged chewing habits among participants. These observations suggest that, in otherwise healthy individuals, moderate chewing may be well tolerated or even advantageous, promoting masticatory muscle conditioning and neuromuscular resilience.

Nevertheless, the consequences of chewing appear to be shaped by its pattern and context. Chewing associated with meals tends to be symmetrical, deliberate, and temporally limited, whereas parafunctional activity—such as habitual or stress-induced gum use—can be prolonged, imbalanced, and unconscious, potentially resulting in repetitive strain to the masticatory apparatus. Such behaviors closely relate to bruxism, a common parafunctional activity involving repetitive clenching or grinding of the teeth, with a multifactorial etiology that includes psychological stress, anxiety, and adverse oral habits [46]. Recent meta-analyses estimate the global prevalence of sleep bruxism at 21% and awake bruxism at 23% [47].

Individuals with bruxism often exhibit increased thickness and activity of the masticatory muscles, which may result in pain, muscle fatigue, and overload of the temporomandibular joints [48]. A strong association has been established between bruxism and TMDs, with both conditions affecting the TMJ and masticatory muscles [49,50]. Shared contributing factors—particularly stress and parafunctional behaviors—may further reinforce this connection [23]. Neuroimaging studies have revealed that central nervous system mechanisms, including cortical and subcortical circuits, play a key role in the initiation and modulation of bruxism episodes [51]. In addition, ultrasonographic findings suggest that individuals affected by both bruxism and TMD often demonstrate significantly greater masticatory muscle hypertrophy compared with non-bruxers, underscoring the close interaction between bruxism and musculoskeletal adaptation [48].

Given this background, other common oral behaviors—such as chronic gum chewing—warrant closer investigation regarding their potential link to TMD development. Determining the threshold at which chewing behavior transitions from adaptive to maladaptive remains a key direction for future research. This nuanced view supports the integration of chewing behaviors into a broader biopsychosocial understanding of TMD pathogenesis, rather than evaluating them in isolation. This contextual interpretation may also help explain inconsistent findings in previous studies regarding the effects of habitual gum chewing.

Taken together, these insights indicate that, while gum chewing alone may not directly cause TMDs, its contribution as a parafunctional habit—particularly when combined with bruxism and psychosocial stressors—can amplify masticatory muscle load and joint strain, thereby increasing the risk of TMD development in susceptible individuals.

### 4.4. Study Limitations

This study has several limitations that should be considered when interpreting the findings. The limited sample size (N = 66) and the homogeneity of the population—consisting solely of medical students from Krakow—limit the generalizability of the results to the wider population [45]. The narrow age range (19–30 years) and the high academic stress exposure may not be representative of other demographic groups. Additionally, the use of a convenience sampling approach may have introduced selection bias.

Chewing behaviors were assessed using a self-reported questionnaire. This introduces the potential for recall bias, especially in estimating frequency, duration, and chronicity of gum use. Another methodological limitation was the inability to conduct multivariate logistic regression due to the small number of TMD-free participants. Consequently, potential confounders such as sex, stress levels, and parafunctional intensity could not be statistically controlled. Finally, the cross-sectional design precludes causal inferences. Future longitudinal studies with larger and more heterogeneous populations are needed to evaluate whether excessive gum chewing contributes to the development or persistence of TMD symptoms over time.

## 5. Conclusions

The results of this study do not support a direct association between excessive gum chewing and the occurrence of TMDs in young adults. While TMD was highly prevalent in the study population, chewing-related behaviors alone did not appear to significantly influence its presence. However, given the cross-sectional design and limited statistical power, these findings should be interpreted with caution. They underscore the multifactorial nature of TMD and highlight the importance of considering broader behavioral and psychosocial factors. Further longitudinal and multi-center studies involving more diverse populations are needed to clarify the potential modulating role of gum chewing in the context of other risk factors such as stress, bruxism, and occlusal disturbances. Although definitive conclusions cannot be drawn, the present findings offer a meaningful contribution to the debate on gum chewing as a potential parafunctional behavior and underscore the complexity of TMD etiology.

## Figures and Tables

**Table 1:** Characteristics of the study group.

Variable		Value
Total number of participants		66
Age [years]	Range	19–30
	Median (IQR)	23 (23–24)
	Mean (SD)	23.44 (1.73)
Sex	Female	41 (62.12%)
	Male	25 (37.88%)

**Table 2:** Distribution of TMD subtypes by gender.

Gender	Participants (n)	TMD Overall (n, %)	Muscle-Related TMD (n)	Joint-Related TMD (n)	Combined TMD (n)
Female	41	35 (85.37%)	7	8	20
Male	25	20 (80.00%)	2	6	12
Total	66	55 (83.33%)	9	14	32

**Table 3:** Detailed results of muscle-related TMD diagnostics (bilateral assessment).

TMD Subtype	Diagnosis	N
Muscle disorders	Myalgia	18
	Arthralgia	1
	Other muscle-related diagnosis	57
No muscle disorder		56

**Table 4:** Detailed results of joint-related TMD diagnostics (bilateral assessment).

TMD Subtype	Diagnosis	N
Joint disorders	Disc displacement with reduction	19
	Disc displacement without reduction with limited opening	1
	Disc displacement without reduction without limited opening	6
	Degenerative joint disease	1
	Other joint-related diagnosis	39
No joint disorder		66

**Table 5:** Distribution of participants by gum-chewing frequency, chewing duration per session, and chronicity of chewing habit.

Variable	Group	Answer	Number of Participants		
Chewing frequency	Non-systematic	Less often	4	37	56.1%
		Once a month	10		
		Several times a month	7		
		Once a week	16		
	Systematic	Several times a week	15	29	43.9%
		Once a day	3		
		Several times a day	11		
Chewing duration	Short chewing	<10 min	30	30	45.5%
	Long chewing	10–30 min	32	36	54.5%
		>30 min	4		
Habit chronicity	Short-term	<1 year	6	19	28.8%
		1–3 years	6		
		3–5 years	7		
	Long-term	>5 years	47	47	71.2%

**Table 6:** Univariate logistic regression results.

Variable	Group	N	TMD	OR	95% CI	*p*
Chewing frequency	Non-systematic	37	31	1 (ref.)	-	-
	Systematic	29	24	0.929	0.253–3.412	0.912
Chewing duration	Short chewing	30	24	1 (ref.)	-	-
	Long chewing	36	31	1.550	0.422–5.693	0.509
Habit chronicity	Short-term	19	17	1 (ref.)	-	-
	Long-term	47	38	0.497	0.097–2.549	0.402

Note: No statistically significant associations were found (all *p* > 0.05).

## Data Availability

The datasets supporting the findings of the current study are available from the corresponding author on reasonable request.
